# Feasibility of a Novel Transcatheter Valve Repair System to Treat Tricuspid Regurgitation in ccTGA

**DOI:** 10.1016/j.jaccas.2021.04.024

**Published:** 2021-06-16

**Authors:** Peter Luedike, Matthias Riebisch, Alexander Weymann, Arjang Ruhparwar, Tienush Rassaf, Amir A. Mahabadi

**Affiliations:** a Department of Cardiology and Vascular Medicine, West German Heart and Vascular Center, University Hospital Essen, University Duisburg-Essen, Essen, Germany; b Department of Thoracic and Cardiovascular Surgery, West German Heart and Vascular Center Essen, University Hospital Essen, University Duisburg-Essen, Essen, Germany

**Keywords:** ccTGA, PASCAL, tricuspid regurgitation, ccTGA, congenitally corrected transposition of the great arteries, MRI, magnetic resonance imaging, NT-proBNP, N-terminal pro–B-type natriuretic peptide, NYHA, New York Heart Association, TEER, transcatheter edge-to-edge repair, TOE, transoesophageal echocardiography

## Abstract

Patients with congenitally corrected transposition of the great arteries (ccTGA) with significant systemic tricuspid valve regurgitation and systemic right ventricular dysfunction have prohibitive surgical risk in adulthood. The PASCAL transcatheter valve repair system enables minimally invasive percutaneous tricuspid valve repair in the complex anatomy of patients with ccTGA. (**Level of Difficulty: Intermediate.**)

## Central Illustration

**Figure:**
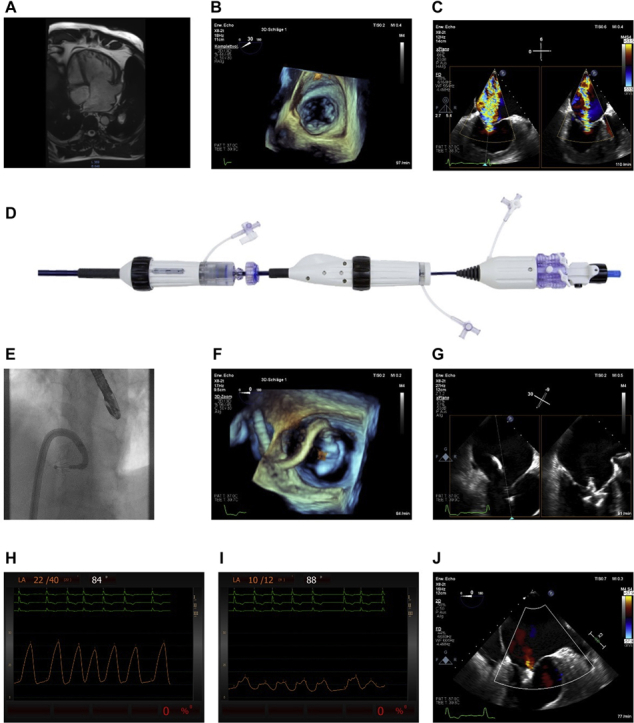

## History of Presentation

We report the first case of a 59-year-old patient with New York Heart Association (NYHA) functional class III due to congenitally corrected transposition of the great arteries (ccTGA) with failing systemic right ventricle and significant, eccentric insufficiency of the tricuspid valve who was treated with the novel PASCAL system. The patient suffered from progressive dyspnea over the previous 3 years, showed decreasing ventricular ejection fraction of the systemic ventricle, and was hospitalized for cardiac decompensation 2 times within 10 months before the intervention reported here.

**Learning Objectives**

• To recognize that interventional TEER is a feasible therapy for patients at prohibitive risk for surgery with ccTGA and severe tricuspid regurgitation.
• To differentiate between existing transcatheter leaflet approximation techniques and their applicability in complex anatomies.

## Past Medical History

The cardiac index of the patient was reduced at 2.18 l/min/m^2^, postcapillary wedge pressure was 21 mm Hg, ejection fraction of the systemic ventricle was 33% (quantified by means of magnetic resonance imaging [MRI]), the vena contracta width was 9 mm, and N-terminal pro–B-type natriuretic peptide (NT-proBNP) level was 2,479 pg/ml. The patient was listed for heart transplantation and presented with progressive dyspnea and severe orthopnea despite optimal medical treatment (5 mg bisoprolol twice daily, 49/51 mg sacubitril/valsartan twice daily, 25 mg spironolactone daily, and torasemide 5 mg daily) at our tertiary heart failure center. After interdisciplinary heart team discussion, transcatheter percutaneous edge-to-edge (TEER) repair of the tricuspid valve was recommended as bridge-to-transplant strategy because of prohibitive risk for surgery.

We received written approval from the patient and the ethics committee of our institution (20-9223-BO) before publication of the case.

## Differential Diagnosis

Patients with ccTGA and significant insufficiency of the atrioventricular valve of the systemic right ventricle commonly develop heart failure in adulthood. Because these patients are at prohibitive risk for surgery, few percutaneous approaches using the TEER approach with existing technologies (MitraClip, Abbott, Abbott Park, Illinois) are described in the literature (1, 2, 3). These percutaneous approaches represent particularly challenging candidates because of their complex anatomy and chronically adopted hemodynamic. Dilation of the left atrium with consecutive reduction of compliance and contractility of the atrium in these patients raise the need for a minimally invasive repair without increasing the pressure gradient across the valve. The recently introduced leaflet repair therapy, the PASCAL transcatheter valve repair system (Edwards Lifesciences, Irvine, California) is a novel concept using clasps and paddles to place a woven nitinol spacer between the native valve leaflets to fill the regurgitant orifice (4,5). This technology is also termed transcatheter edge-to-spacer repair technique. The combination of the central spacer coupled with broad and contoured paddles is designed to reduce stress on the leaflets, which is supposed to translate into reduced pressure gradients across the valve despite being as big as the largest available TEER device. The ability to span large vena contracta gaps while not creating high pressure gradients predestinates this new technology for transcatheter leaflet approximation in the particular anatomy of ccTGA patients. Because this recently launched system offers, in addition to the favorable hemodynamic consideration, a flexible steering technology that allows for free mobility on all levels without losing precision, it seems to be well suited to treat these patients, which has not been reported in the literature.

## Investigations

**Figure 1:**
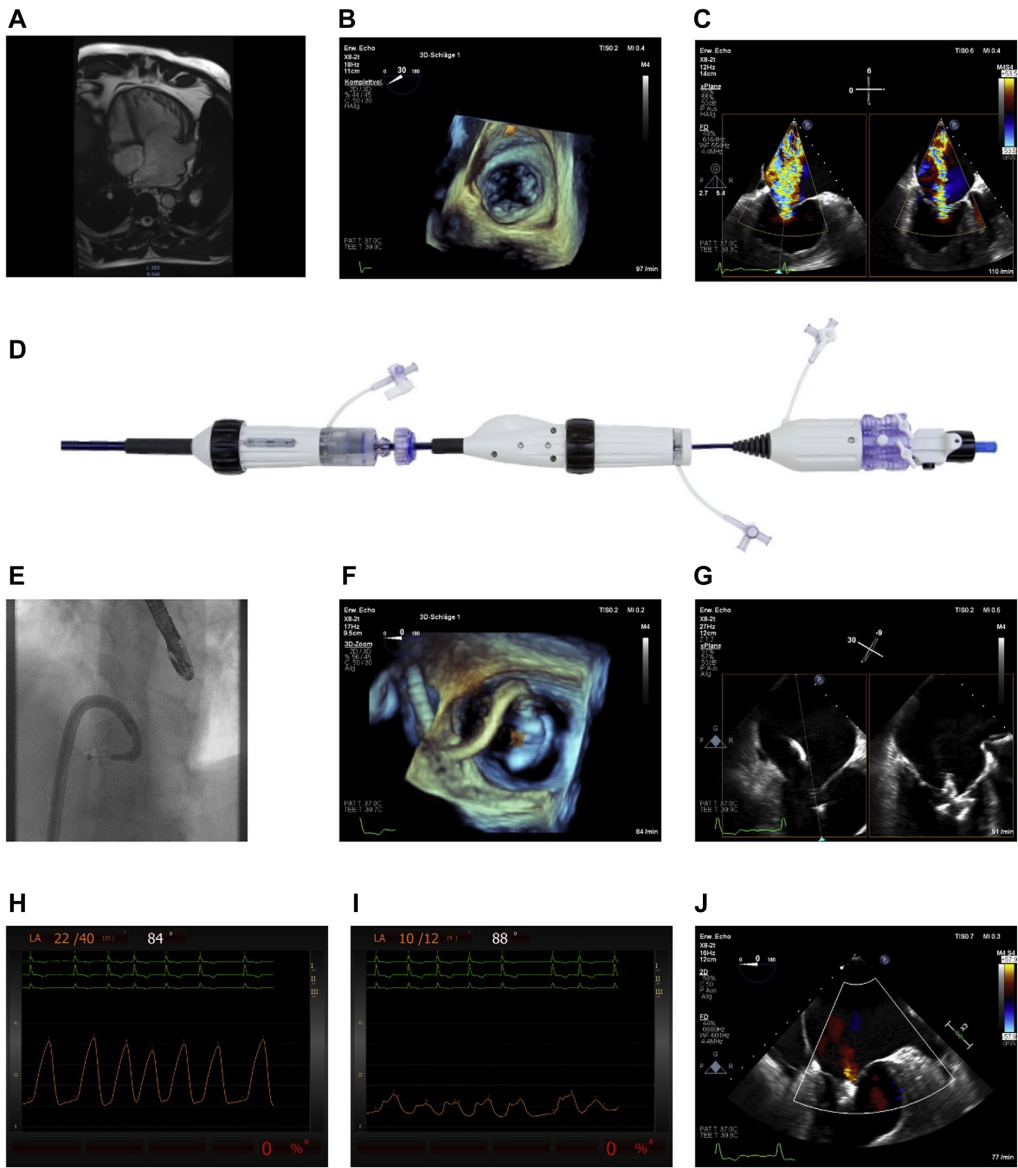
Transcatheter Edge-to-Spacer Repair of a Tricuspid Valve in a Case of Congenitally Corrected Transposition of the Great Arteries **(A)** 4-chamber view of cardiac magnetic resonance imaging shows the dominance of the right ventricle and the anterior shifting of the tricuspid annular plane. **(B)** Pre-procedural 3-dimensional (3D) transoesophageal echocardiography (TOE) shows 3 independent leaflets with typical anatomic allocation (septal, posterior, anterior). **(C)** 2-dimensional (2D) imaging (x-plane) of the main regurgitation jet in the posteroseptal commissure. **(D)** Design of the device with three embedded independent catheters and steering knobs (**left to right**: guide sheath handle, steerable catheter handle, implant catheter handle). **(E)** Fluoroscopic illustration of extreme angulation of the guide sheath and the steerable catheter (left anterior oblique 25°, cranial 30°). **(F)** 3D-guided steering. **(G)** 2D illustration of independent leaflet grasping (x-plane). After interventional valve repair, the V-wave was reduced from 40 mm Hg to 12 mm Hg **(H, I)** and regurgitation was reduced to a trace **(J)**.

Pre-procedural imaging included cardiac MRI (Figure 1A) on top of transesophageal echocardiography (TOE) because standardized views were not available in the given anatomy. TOE revealed that the valve of the systemic ventricle had 3 leaflets with physiologic anatomic allocation (septal, posterior, anterior) (Figure 1B). The primary insufficiency jet in this patient was located in the posteroseptal commissure of the tricuspid valve owing to tethering of the septal leaflet (Figure 1C). Because adequate capture of the leaflets during TEER necessitates a minimum distance of 36 mm between leaflet coaptation and height of the transseptal puncture, this measurement was also confirmed in cardiac MRI on top of TOE analysis. Preinterventional MRI revealed that the membranous part of the interatrial septum was high enough (42 mm) above the tricuspid valve annulus for transseptal puncture. The enlargement of the systemic right ventricle led to a shift of the tricuspid valve area in the sagittal plane toward the sternum. These anatomic circumstances necessitated an extreme maneuvering of the steering system, with a 90° angulation of the guide sheath (clockwise rotation of the flex knob of the guide sheath handle), a 90° angulation (clockwise rotation of the flex knob of the steerable handle), and a counterclockwise rotation of the steerable catheter (by rotation of the whole steerable handle). Because no standardized TOE views were available, steering was controlled in the 3-dimensional view (Figures 1D to 1F).

## Management

Despite the extreme angulation, steering and maneuvering were not restricted. After adaptation of the posterior and septal leaflets with the use of several independent grasping attempts (Figure 1G), tricuspid insufficiency was reduced from torrential to trace (Figure 1J). Reduction of tricuspid regurgitation was paralleled by a reduction of the v-wave from 40 mm Hg to 12 mm Hg immediately after release of the device (Figures 1H and 1I). The mean pressure gradient across the valve remained unchanged at 1 mm Hg.

## Discussion

The novel PASCAL technology is available for both mitral-and tricuspid valve repair demonstrating the flexibility of the system, while recently a dedicated TriClip system was introduced as an advancement of the existing MitraClip platform for meeting the needs of complex steering in the right ventricle. Reports on the TriClip device for the treatment of tricuspid insufficiency in ccTGA do not exist yet. Because no head-to-head comparison of the existing transcatheter leaflet approximation techniques exists and interventional tricuspid valve repair in ccTGA is a rarely performed procedure, it remains speculative whether one technique is superior to the other when treating these complex anatomies.

## Follow-Up

Dyspnea of the patient improved immediately after the procedure (NYHA functional class I). NT-proBNP decreased from 2,479 pg/ml to 1,587 pg/ml, and the patient was discharged 4 days after the procedure and reported NYHA functional class I.

## Conclusions

This is the first report of percutaneous tricuspid valve repair using a novel TEER system in a patient with ccTGA. This first experience demonstrated that the steering mechanism allows for optimal maneuvering in complex anatomies and that patients with severe tricuspid regurgitation in ccTGA can be safely treated with this technology without significant increase of the mean pressure gradient across the valve.

## Funding Support and Author Disclosures

Dr. Luedike has received a research grant and research, consulting, and speaker honoraria from Edwards Lifesciences. All other authors have reported that they have no relationships relevant to the contents of this paper to disclose.
